# Effect of Patient Support Groups for Hypertension on Blood Pressure among Patients with and Without Multimorbidity: Findings from a Cohort Study of Patients on a Home-Based Self-Management Program in Kenya

**DOI:** 10.5334/gh.1208

**Published:** 2023-06-09

**Authors:** Peter Otieno, Charles Agyemang, Calistus Wilunda, Richard E. Sanya, Samuel Iddi, Welcome Wami, Judith Van Andel, Babette van der Kloet, Julia Teerling, Angela Siteyi, Gershim Asiki

**Affiliations:** 1 African Population and Health Research Center P.O. Box: 10787-00100, Nairobi, Kenya; 2 Department of Public & Occupational Health, Amsterdam UMC, University of Amsterdam, Amsterdam Public Health Research Institute, Amsterdam, The Netherlands; 3 Amsterdam Institute for Global Health and Development (AIGHD), AHTC, Tower C4, NL; 4 PharmAccess Foundation, Nairobi, Kenya; 5 Department of Women’s and Children’s Health, Karolinska Institutet, Stockholm, Sweden

**Keywords:** patient support groups, home-based self-care, hypertension, multimorbidity, blood pressure

## Abstract

**Introduction:**

Patient support group interventions have been widely used to manage chronic diseases in Kenya. However, the potential benefits of these groups on patient health outcomes, and how this is influenced by multimorbidity, have not been rigorously evaluated.

**Objective:**

We assessed the effect of a patient support group intervention on blood pressure (BP) management and the potential moderating effect of multimorbidity among low- and middle-income patients with hypertension in Kenya.

**Methods:**

We analysed data from a non-randomized, quasi-experimental study of 410 patients with hypertension on a home-based self-management program conducted from September 2019 to September 2020. The program included the formation and participation in patient support groups. Using a modified STEPS questionnaire, data were collected on BP, anthropometry and other measurements at enrolment and after 12 months of follow-up. Multimorbidity was defined as the simultaneous presence of hypertension and at least one or more related conditions with similar pathophysiology (concordant multimorbidity) or unrelated chronic conditions (discordant multimorbidity). Propensity score (PS) weighting was used to adjust for baseline differences among 243 patients who participated in the support groups and 167 who did not. We estimated the effects of patient support groups and moderating effects of multimorbidity on BP management using multivariable ordinary linear regression weighted by PS.

**Findings:**

Participation in support groups significantly reduced systolic BP by 5.4 mmHg compared to non-participation in the groups [β = –5.4; 95% CI –1.9 to –8.8]. However, among participants in the support group intervention, the mean systolic BP at follow-up assessment for those with concordant multimorbidity was 8.8 mmHg higher than those with no multimorbidity [β = 8.8; 95% CI 0.8 to 16.8].

**Conclusion:**

Although patient support groups are potentially important adjuncts to home-based self-care, multimorbidity attenuates their effectiveness. There is a need to tailor patient support group interventions to match the needs of the people living with multimorbidity in low- and middle-income settings in Kenya.

## Background

Hypertension is the leading global risk factor for cardiovascular disease (CVD) [1,2]. Low- and middle-income countries (LMIC) are disproportionately affected with over 80% of global CVD deaths [3]. In Kenya, one-in-four adults live with hypertension [2]. However, less than half of people on treatment for hypertension have controlled blood pressure (BP) [4,5]. Management of hypertension is a complex process requiring collaborative efforts of the patients, the health sector, and wider society [6,7,8,9,10,11]. The World Health Organization (WHO) proposes peer support groups as an intervention to promote patients’ coping behaviour, psychosocial functioning, medication adherence, and retention in care [12]. A patient support group comprise a group of patients sharing common experiences and concerns and who provide moral and emotional support to each other by fulfilling functions such as health education and behaviour change communication, public awareness, health advocacy, and fundraising [13].

Patient-led support groups represent an ideological shift away from patients as ‘passive’ recipients of treatment to empowered individuals who are partners in the effective management of their health [14]. In Kenya, patient support group interventions have been widely used [15,16,17,18]. However, their impacts have not been systematically evaluated. A study by Pastakia et al. conducted in 2017 demonstrated the success of a patient support group intervention in helping to improve care for hypertension in rural settings in Kenya [18]. However, this study did not incorporate the impact of multimorbidity on the self-care intervention. People living with hypertension often have multiple rather than a single condition, also known as multimorbidity [19]. One in every two people with hypertension has a multimorbidity [20]. Despite the potential implications of multimorbidity on the effectiveness of patient support groups [21], existing interventions have not adequately incorporated its impact on health outcomes [21]. Hence, it is not possible to determine whether the interventions are particularly effective for people living with multimorbidity. Given the rising prevalence of multimorbidity in Kenya [22,23], it is important to understand the effects of multimorbidity on patient support group interventions to inform on the appropriate models to deploy.

In this study, we registered patients from low- and middle-income settings in Kenya and provided access to self-management tools such as blood pressure devices to help them with self-measurements at home. They were also provided with mobile phone applications to relay their measurements to primary clinics via their mobile phones. The patient support groups were introduced during the follow-up period to improve the uptake of self-measurements. Secondary data analysis was used to evaluate the moderating effects of multimorbidity on the effectiveness of patient support groups. We hypothesized that multimorbidity would moderate the effectiveness of patient support group intervention among low and middle-income patients in Kenya.

## Methods

### Study design

We analysed data from a nonrandomized, quasi-experimental pilot study of hypertension patients undergoing a home-based self-care program from September 2019 to September 2020. Therefore, we utilized inverse probability of treatment weighting using propensity scores (IPTW-PS) to create a comparison (non-exposed) group which was similar to the exposed group on all measured covariates except for the exposure. The propensity score (PS) is defined as the probability of being in the intervention group conditional on the observed participant’s baseline characteristics [24]. IPTW-PS is a statistical approach that weights the exposed and nonexposed groups using PS. Thus minimizing the selection bias and confounding.

### Study setting

The study population included patients seeking healthcare services from facilities serving low- and middle-income populations from three Kenyan Counties: Nairobi, Kiambu, and Vihiga. These facilities were selected because they were involved in a chronic disease care program called *Ngao Ya Afya-Tiba Yako*. The program was supported by the African Population and Health Research Center (APHRC) and the PharmAccess Foundation. Nairobi, the capital city of Kenya is the most populous county and represents an urban metropolitan setting [25]. Kiambu County is the second most populous county after Nairobi and represents a semi-urban setting while Vihiga represents a rural setting [25,26]. The three counties included in this study are in different geo-political areas. The inclusion of these three counties accounted for the variations in the burden of hypertension and lifestyle risk factors in different geographical and social contexts.

### Participant recruitment

Participants were recruited from June 2019 to September 2019 and followed up for one year (September 2019 to September 2020). Known and new patients with essential hypertension who were receiving care at one of the study clinics were invited to participate in the study. To recruit new patients, screening was performed at clinics during triage for regular visits. The inclusion criteria comprised, (i) patients with a new diagnosis of essential hypertension (diagnosis made by treating clinician), (ii) patients known to have essential hypertension (diagnosis made by treating physician) who were already receiving medication, (iii) patients with intervention receiving intervention provided by the recruiting site, (iv) adult (>18 years old), and (vi) ownership of a mobile phone.

The exclusion of the study participants was based on seven criteria: (i) patients with suspected secondary hypertension from the assessment of the treating physician acting in accordance with the clinical guidelines, (ii) patients requiring intervention (secondary, tertiary hypertension care) not provided by the recruiting site, (iii) arm circumference greater than or less than the 22–42 cm for which the used cuffs are validated, (iv) failure to obtain valid BP-values (e.g. cardiac arrhythmias), (v) pregnancy, (vi) unsuitability for receipt of mobile hypertension care as judged by the treating physicians (for instance, patients with life-threatening diseases or dementia), and (vii) an acute cardiovascular event in the past three months preceding the survey.

### Sample size

Given that the uptake of patient support group intervention from the original study was 60%, a sample size of 465 participants, 278 in the intervention group and 187 in the control group was required to reject the null hypothesis that BP control was equal in the intervention and control groups [27]. This provides 80% power to detect a 15% increase in BP control in the support group intervention compared to the control group assuming a 5% level of significance (two-sided test) and a non-response rate of 20%. However, baseline and follow-up data were available for 410/465 participants. Thus the response rate was 243/278 (87.4%) in the intervention arm and 167/187 (89.3%) in the control arm.

### Description of the intervention

The intervention included a home-based self-care program and patient support groups. All the 410 recruited participants were enrolled in the home-based self-management program. The control group received the home-based care program only while the intervention group received home-based care program and the patient support group intervention.

#### Home-based self-management

**Figure 1:**
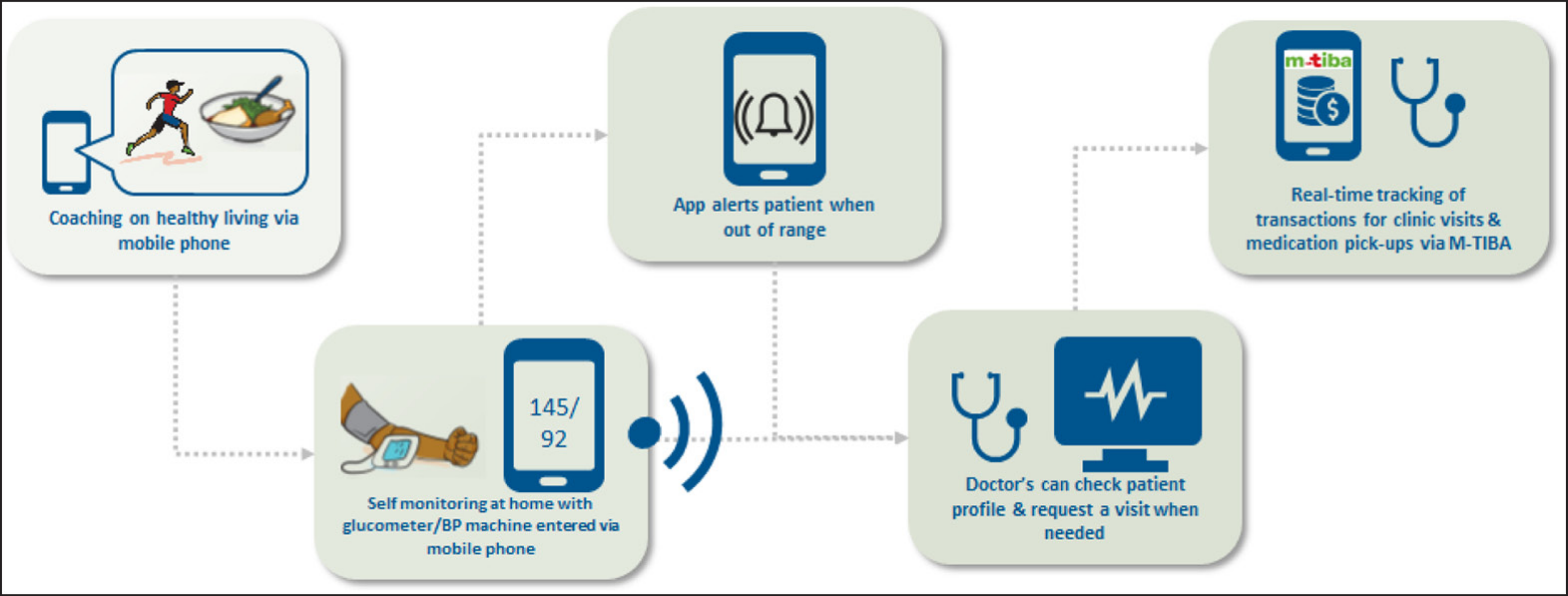
Care model for home measurement of blood pressure.

Self-management devices (BP machines) were distributed to all the participants to measure BP at home. Figure 1 shows the care model for home-based measurement of BP. All participants were trained to take their measurements at home and enter their readings on a mobile phone application (*Afya Pap*), to relay their measurements to their healthcare provider. Further details about the *Afya Pap* application are available elsewhere [28]. In addition, health education messages were sent to all the enrolled patients through the *Afya Pap* application. Finally, all the participants were also enrolled in a mobile health wallet (*M-TIBA*) that gives access to discounts on consultations, medical tests for hypertension and medicines at the study clinics [29].

#### Patient support groups

The support group intervention involved formation of patient support groups and comprised four components: peer-led training, peer support for home-based self-care and lifestyle modification, BP measurement demonstrations, and group measurements. For the peer-led training, leader volunteer members of patient support groups participated in the training of peers through face-to-face health coaching and information support for self-management of hypertension, emotional support using motivation-counselling techniques and appraisal support using self-management skills. A clinical officer at the health facility attached to the peer group conducted BP measurement demonstrations. The clinical officer ensured that the group members were well equipped with the necessary knowledge of hypertension management and peer leadership skills. In the support groups, the patients participated in group measurement of BP and exchanged knowledge and experiences on hypertension self-management and healthy lifestyles.

Participants were invited to join these facility-based patient support groups which met monthly. In total 243/410 individuals joined and participated in groups. Each group had an average of 32 members. For purposes of understanding whether the patient support groups augmented the home-based self-management intervention, participants were grouped into two categories: those who joined the patient support groups (n = 243) and those who did not (n = 167).

### Data collection

We used a modified WHO STEPwise approach to non-communicable disease risk factor surveillance (STEPS) questionnaire to collect data at enrolment and after 12 months of follow-up. The details of the measurements of variables used in this study are shown in the online supplementary file 1. The interview questions consisted of socio-demographic characteristics (age, sex, and employment), CVD risk factors (physical activity, use of alcohol, smoking status, and healthy diet), medication adherence, and frequency of self-measurement of BP. The variables were measured using the WHO criteria [30]. Briefly, the history of smoking tobacco products was self-reported and defined as a current smoker. Physical activity was measured as the average days of planned physical activity in a week. Diet was measured as the self-reported daily number of servings of fruits and vegetables. Medication adherence was measured as the average number of days the patients took hypertension medicine in the week preceding the survey.

The diagnosis of hypertension was made following an objective assessment by the attending clinician. Multimorbidity was assessed by screening and self-reports. Patients were screened for type 2 diabetes during regular clinic visits and the diagnosis made by the attending clinician. Self-reports were used to document the presence of other chronic diseases such as CVD, hypercholesterolemia, chronic kidney disease, asthma, arthritis, chronic neuromuscular disease, HIV/AIDS, tuberculosis, cancer, ulcers, depression, chronic liver disease, and depression. Weight (in kg) and height (in metres) were also measured. Since the patients enrolled in this study had BP measuring devices previously issued at enrolment, they were requested to take their BP measurements at the time of the follow-up interview and relay the information to the interviewer via short message texts. All the data were electronically captured on tablets using the Survey CTO platform (Dobility, Inc. Cambridge, USA), synchronized with the master database and exported to Stata version 17.0 (StataCorp LP, USA) for analysis.

### Definition and measurement of variables

The primary outcome was endline mean systolic and diastolic BP. The explanatory variables were participation in the patient support group (intervention), multimorbidity status, and interaction of multimorbidity with the intervention arms. Multimorbidity was defined as the simultaneous presence of hypertension and one other condition with related pathophysiology i.e., type 2 diabetes, CVD, obesity, hypercholesterolemia, chronic kidney disease (concordant multimorbidity), and unrelated conditions such as asthma, arthritis, chronic neuromuscular disease, HIV/AIDS, tuberculosis, cancer, ulcers, depression, chronic liver disease, and depression (discordant multimorbidity). Participants were classified into the following four mutually exclusive multimorbidity categories: no multimorbidity, concordant multimorbidity, discordant multimorbidity, and both concordant and discordant multimorbidity. Other covariates were age, sex, occupation, smoking, alcohol, diet, medication adherence, and baseline BP.

### Data analysis

#### Propensity score weighting

We used PS weighting [31] to adjust for baseline differences in participation in the peer support groups. The PS scores were generated using a multivariable logistic regression model, with participation in peer support group as the outcome variable and the following baseline characteristics as predictors: age, sex, employment, diet, physical activity, medication adherence, and BP control. We used the estimated PS to weight the groups, with the exposed group weighted using the inverse of PS (1/PS) and the comparison group weighted using the inverse of one minus the PS (1/(1 – PS)). This created a pseudo-population with balanced covariates. Baseline categorical data were summarized using frequencies, percentages, and numerical data using means with standard deviation. Group comparisons comprising paired sample t-test for continuous variables, McNemar’s Chi-squared test, and marginal homogeneity test for categorical variables were used to test the differences in the baseline characteristics by study arms.

#### Regression analysis

We estimated the moderating effects of multimorbidity on BP using multivariable ordinary linear regression with robust error variances, weighted by PS. The primary outcome was regressed against dummy variables, indicating whether the participant participated in the patient support groups, multimorbidity status and interaction of patient support with multimorbidity status. The interaction can be interpreted as a test of whether the difference between intervention and control patients was the same by multimorbidity status. Other covariates included in the model comprised baseline characteristics such as age, sex, employment, diet, smoking, alcohol, physical activity medication adherence, and baseline BP. Variable selection for the multivariable models was based on known risk factors for hypertension [32]. The intervention effect was assessed using adjusted β coefficients (mean differences) and 95% confidence intervals (CIs). The margins and *margins plot* command in Stata was used to graph the output from the predictive margins of significant interactions.

## Results

### Baseline characteristics of the participants

In total, 410 participants were included in the analysis. Table 1 shows no significant differences in the study arms by baseline characteristics. The weighted sample comprised the intervention arm with 243 patients who participated in the peer support group and a control arm with 167 patients who did not participate in the peer support groups.

**Table 1:** Baseline characteristics of the participants.


BASELINE CHARACTERISTICS	PATIENT SUPPORT GROUP		

	INTERVENTION N = 243	CONTROL N = 167	STD. DIFF

Age, Mean ± SD	57.3 ± 11.4	58.1 ± 11.7	0.1

Sex			

Male	30.5	31.1	0.0

Female	69.6	68.9	

Employment			

Employed	58.0	52.7	0.1

Unemployed	42.0	47.3	

Smoking	1.2	4.2	0.2

Alcohol use	3.3	4.2	0.1

Adequate diet	63.8	59.9	0.1

Medication adherence	86.0	88.0	0.1

Average days of physical activity in a week ± SD	2.2 ± 2.2	2.3 ± 21.2	0.0

Multimorbidity			

Type 2 diabetes	39.5	42.0	0.0

Obesity	43.7	39.9	0.1

CVD	16.7	9.1	0.2

Arthritis	6.6	7.0	0.0

Asthma	3.6	4.1	0.0

Chronic Kidney Disease	3.6	2.1	0.1

Tuberculosis	3.0	1.7	0.1

Cancer	3.6	1.7	0.1

Chronic neuromuscular disease	2.4	1.2	0.1

HIV/AIDS	0.6	1.2	0.1

Ulcers	1.2	1.2	0.0

Depression	0.0	0.8	–

Chronic liver disease	0.6	0.4	0.0

Cataract	0.0	0.4	–

Hypercholesterolemia	4.6	0.0	–

Multimorbidity type			0.1

No multimorbidity	26.8	25.2	

Both concordant & discordant multimorbidity	10.3	14.4	

^†^Concordant multimorbidity	57.6	55.7	

^‡^Discordant multimorbidity	5.4	4.8	

Systolic BP ± SD	136.5 ± 19.2	139.0 ± 20.7	0.1

Diastolic BP ± SD	87.8 ± 12.7	89.5 ± 12.3	0.1

BP Control	46.9	45.5	0.0


*Notes*: Data presented as column %, unless otherwise specified. BP: blood pressure; SD: standard deviation; Std Diff: standardized difference. Std Diff = Difference in means or proportions divided by standard error; imbalance defined as an absolute value greater than 0.2. ^†^ Concordant multimorbidity refers to conditions with shared pathophysiology such as type 2 diabetes, CVD, obesity, hypercholesterolemia, and chronic kidney disease. ^‡^ Discordant multimorbidity refers to conditions with unrelated pathophysiology such as asthma, arthritis, chronic neuromuscular disease, HIV/AIDS, tuberculosis, cancer, ulcers, depression, chronic liver disease and depression.

### Changes in lifestyle risk factors and BP by intervention arms

Table 2 shows the changes in lifestyle risk factors and BP by intervention arms. Physical activity, frequency of self-measurement of BP, and consumption of adequate diet increased substantially during follow-up in the intervention and control arms. Medication adherence declined slightly in both study arms from 88.0% to 83.5% in control and 86.0% from 83.5% in the intervention arm. There was a slight increase in alcohol consumption in both study arms. However, substantial smoking decline was observed in the control arm but not in the intervention arm. The proportion of controlled BP among patients in the intervention arm significantly increased from 44.9% to 57.6% compared to a slight increase from 45.5% to 46.1% in the control arm. The mean BP reduced marginally among patients in the intervention arm (from 136.5/87.9 mmHg at baseline to 133.0/85.8 mmHg at end line). There was no significant change in the systolic BP in the control arm. However, the mean diastolic BP reduced marginally (from 90 mmHg at baseline to 87 mmHg at endline).

**Table 2:** Changes in lifestyle risk factors and BP by intervention arms.


		PEER SUPPORT GROUPS					

		INTERVENTION (N = 243)			CONTROL (N = 167)		
	
		BASELINE	FOLLOW-UP	P VALUE*	BASELINE	FOLLOW-UP	P VALUE*

Adequate diet							

	Yes	36.2	55.1	<0.001	40.1	56.9	<0.001

Smoking							

	Yes	1.2	0.8	0.65	4.2	0.0	0.01

Alcohol use							

	Yes	3.3	6.2	0.05	4.2	7.8	0.08

Days of planned physical activity in a week, Mean ± SD							

		2.2 ± 2.2	3.1 ± 2.5	0.00	2.3 ± 2.2	2.7 ± 2.4	0.07

Medication adherence							

	Yes	86.0	83.5	0.24	88.0	83.8	0.09

Frequency of self-measurement of BP							

	Never	47.7	0.8	<0.001	53.3	3.0	<0.001

	Daily	5.4	32.9		4.2	28.1	

	Weekly	11.5	63.0		12.6	67.1	

	Monthly	35.4	3.3		29.9	1.8	

Systolic BP, Mean ± SD							

		136.5 ± 19.2	133.0 ± 15.2	0.01	139.0 ± 20.7	138.8 ± 19.5	0.91

Diastolic BP, Mean ± SD							

		87.9 ± 11.7	85.8 ± 10.5	0.01	89.5 ± 12.3	87.0 ± 11.2	0.01

BP control							

	Yes	46.9	57.6	0.01	45.5	46.1	0.89


*Notes*: Data presented as column %, unless otherwise specified. BP: blood pressure; SD: standard deviation. * P-values for paired sample t-test for continuous variables, McNemar’s Chi-squared test, and marginal homogeneity test for categorical variables.

### Effect of the intervention on blood pressure at follow-up assessment

**Figure 2:**
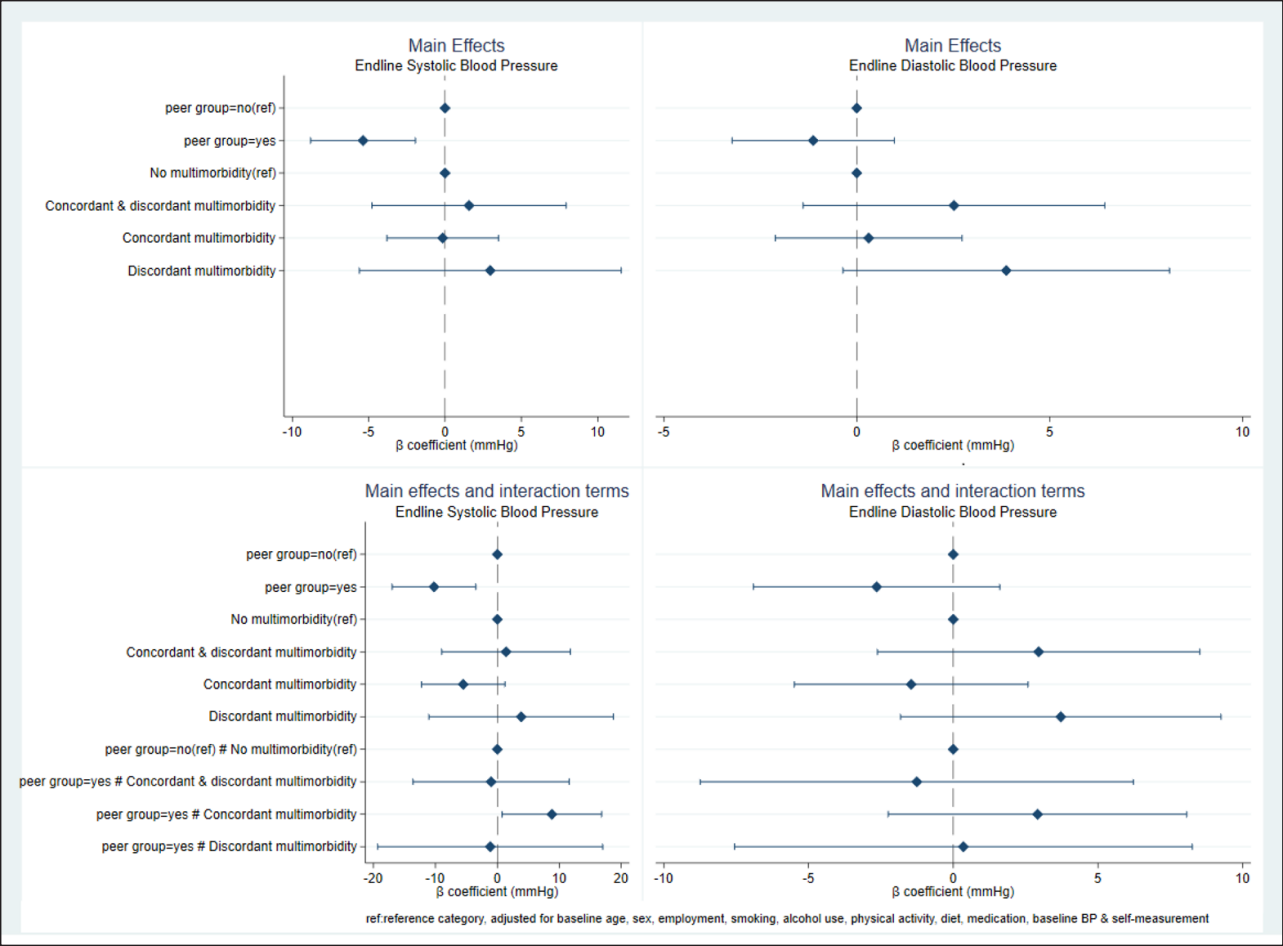
Effect of the intervention on blood pressure at follow-up assessment.

Figure 2 shows the effect of the intervention on BP at the follow-up assessment, moderated by multimorbidity. Participation in support groups significantly reduced systolic BP by 5.4 mmHg compared to non-participation in the groups [β = –5.4; 95% CI –1.9 to –8.8]. A significant interaction was observed between participation in a patient support group and concordant multimorbidity in their effects on BP management. Among participants in the support group intervention, the mean systolic BP at follow-up assessment for those with concordant multimorbidity was 8.8 mmHg higher than those with no multimorbidity [β = 8.8; 95% CI 0.8 to 16.8]. The main effect of patient support groups on diastolic BP was not significant [β = –1.1; 95% CI –3.2 to 1.0]. Similarly, the interaction effect of patient support groups with multimorbidity was also not significant for diastolic BP [β = –2.6; 95% CI –6.9 to 1.6].

### Predicted marginal effects of the intervention on BP at the follow-up assessment, moderated by multimorbidity

**Figure 3:**
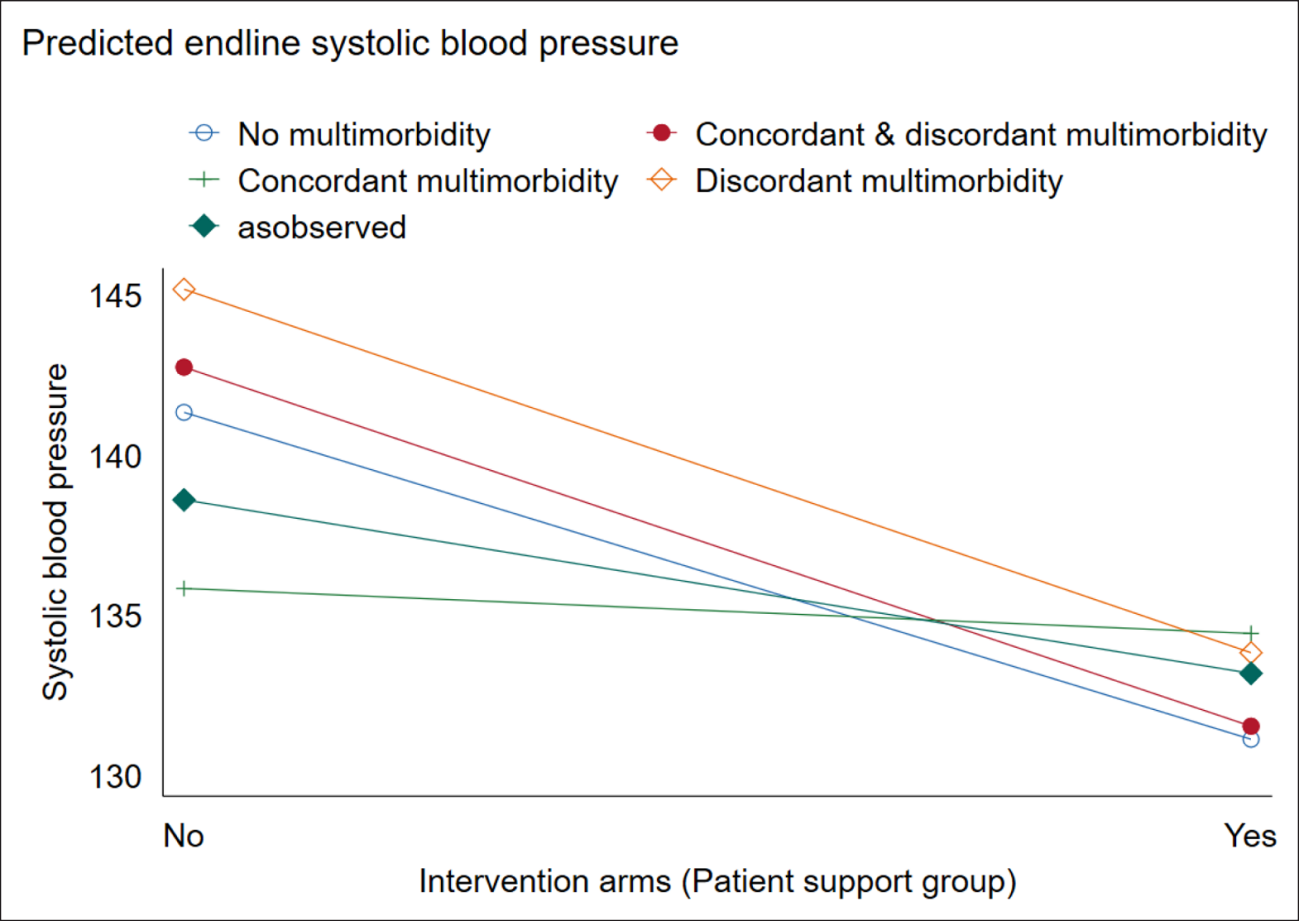
Predicted endline systolic blood pressure.

Figure 3 shows the predicted marginal effects of the intervention on BP at the follow-up assessment, moderated by multimorbidity. The examination of the interaction plot for the predicted marginal effects shows that participation in the patient support groups conferred significantly lower predicted mean systolic BP among participants without multimorbidity than those with multimorbidity.

## Discussion

In this study, we assessed whether the benefits of a patient support group intervention for hypertension in low and middle-income settings in Kenya varied by the presence of multimorbidity. Our results showed that participation in support groups significantly reduced systolic BP compared to non-participation in the groups. However, participation in support groups had a better effect on patients with no multimorbidity as shown by a significant reduction in systolic BP among patients with no multimorbidity compared to those with concordant multimorbidity. These findings confirmed our hypothesis that multimorbidity attenuates the effectiveness of patient support group intervention.

A possible explanation for patient support groups as an important adjunct to home-based self-care could be due to in part the improvement in compliance, as patients become more involved in their care [33,34,35,36]. Our study shows that the patient support group intervention was less effective in patients with concordant multimorbidity. The mechanisms by which multimorbidity affects patient support group interventions among hypertensive patients are unclear. We found two studies with contrasting suggesting that multimorbidity is either a threat or an opportunity for self-care [37,38].

The study by Kerr et al. shows that patients with multimorbidity are more likely to report poor health outcomes from self-care behaviours [37]. People living with multimorbidity face a number of self-care challenges such as limited resources, attention and complex decision-making on self-management priorities. This may impede the self-care behaviours necessary for hypertension management. For example, patients with multimorbidity often have complex intervention regimens with little or no coordination between healthcare services for different conditions. Secondly, therapeutic interventions for multimorbidity are a major challenge due to polypharmacy, drug-disease interactions, and drug-drug interactions [39]. In addition, management of dominant multimorbidity that poses an immediate threat to life such as chronic kidney disease often shifts away the focus from other pre-existing chronic conditions [37]. Worthy of mention also is that failure to find significant interactions between discordant multimorbidity and BP management may a due to the fact that tests for interaction often have limited power [40].

The study by Voorham et al. contrasts our findings and demonstrates that patients with concordant multimorbidity are more likely to report favourable health outcomes from self-care behaviours [38]. Concordant multimorbidities such as type 2 diabetes and CVD share overall pathophysiologic profile and care management plans with hypertension. For example, BP and BG self-monitoring are overlapping CVD risk reduction goals for hypertension and type 2 diabetes and are likely to lead to better health outcomes for both conditions [38]. However, this study was conducted among type 2 diabetes patients in the Netherlands and can be an underestimation of all actual problems or events that may compete with chronic disease management in hypertension patients living with multimorbidity in low- and middle-income settings. Thus, more rigorous studies with large samples are needed to assess the variations in the benefits of self-care interventions by multimorbidity types in Sub-Saharan Africa.

Overall, our findings imply that concordant multimorbidity attenuates the effectiveness of patient support group intervention among low- and middle-income patients in Kenya. The results of this study may contribute to the design of future patient support group interventions, to address the needs of hypertension patients with multimorbidity. The finding of a stronger program effect among hypertensive patients without multimorbidity may help to explain why previous support group interventions sometimes have worked and sometimes have not. Support group interventions for hypertension are more effective when delivered to populations with a low prevalence of concordant multimorbidity. However, more studies are needed to identify the mechanism that underlie poor health outcomes from support group interventions among hypertensive patients with concordant multimorbidity in low and middle-income settings.

### Strengths and limitations

Our study has several strengths. First, we used a quasi-experimental, longitudinal study design to examine whether the benefits of a patient support group intervention for hypertension in low- and middle-income settings in Kenya varied by the presence of multimorbidity. This has enhanced the external validity of the original intervention and the degree to which the findings can be applied to the underserved populations exhibiting high levels of multimorbidity in Kenya. Second, the screening and diagnosis of hypertension and type 2 diabetes multimorbidity were based on an assessment by the treating clinician. This provided for a more objective assessment rather than the self-reporting used in over three-quarters of previous studies [41]. Third, the use of PSM [31] accounted for the conditional probability of participation in the patient support groups, thus allowing for a reduction of bias when examining the effect of support groups on BP management.

These findings need to be interpreted in the context of some inherent limitations. First, the results are based on a post hoc analysis and are clearly in need of replication in future trials. Second, recruitment clinics were not considered as cluster units and thus recruited any number of patients leading to a wide variation in the distribution of patients in the clinics and clinician practices. Third, the screening questions for multimorbidity were partially based on self-reports. This may have resulted in the underestimation of the true prevalence of multimorbidity. Lastly, the multimorbidity classification used in the analysis considered whether the patients had concordant or discordant multimorbidity. Hence, the moderation effect of specific multimorbidity combinations was not explored due to the small sample size. Despite these limitations, our findings provide crucial evidence on the effects of patient support groups and moderating effects of multimorbidity on BP management among low and middle-income patients in Kenya.

## Conclusions

We found evidence that patient support groups can help with reduction in systolic BP among patients with hypertension in low- and middle-income settings in Kenya. However, the findings demonstrate less effectiveness in patients with concordant multimorbidity compared to those without multimorbidity. Thus, tailoring patient support group intervention to match the needs of the people living with concordant multimorbidity may optimize their efficacy. More rigorous cluster randomized trials and operational lessons are needed to maximize the benefits of support groups as an integral component of home-based self-care for hypertension.

## Data Accessibility Statement

The datasets used in this study are available upon a reasonable request to the African Population and Health Research Center (APHRC) through its Microdata portal (https://microdataportal.aphrc.org/index.php/catalog/124).

## Additional File

The additional file for this article can be found as follows:

10.5334/gh.1208.s1 Online supplementary file 1. Measurements of study of the variables.

## Abbreviations

APHRC: African Population and Health Research Center

BP: Blood Pressure

CI: Confidence Intervals

CVDs: Cardiovascular Diseases

IPTW-PS: Inverse Probability of Treatment Weighting using Propensity Scores

LMIC: Low- and Middle-Income Countries

PS: Propensity Score

STEP: STEPwise approach to non-communicable disease risk factor surveillance

WHO: World Health Organization
