# Supplementary material for: Effect of Patient Support Groups for Hypertension on Blood Pressure among Patients with and Without Multimorbidity: Findings from a Cohort Study of Patients on a Home-Based Self-Management Program in Kenya
Source: Glob Heart. 2023 Jun 9;18(1):28. doi: 10.5334/gh.1208 (PMC10253234; doi:10.5334/gh.1208)
Supplement: Online supplementary file 1. — Measurements of study of the variables. [file gh-18-1-1208-s1.pdf]

## List of supplementary files

*Online supplementary file 1: measurements of study of the variables*

| Characteristics | Measurement |
|---|---|
| Age | Mean (Years) ± Standard deviation |
| Sex | Male, Female |
| Employment | Unemployed, Employed |
| Smoking | Current smoking, (Yes, No) |
| Alcohol use | Current alcohol consumption, (Yes, No) |
| Adequate diet | Yes≥ 5 servings per day (fruits or vegetables), No=otherwise |
| Medication adherence | The average number of days the patients took hypertension medicine in the week preceding the survey (mean ± SD), Yes=7/7 days, No=otherwise |
| Frequency of self-measurement of BP in the past month | Never, Daily, Weekly, Monthly |
| Days of planned physical activity in a week | ± Standard deviation |
| BP | mmHg ± Standard deviation |
| BP Control | Yes= SBP <140mmHg and DBP <90 mmHg, No=otherwise |
| BP Control | Yes= SBP <140mmHg and DBP <90 mmHg, No=otherwise |
| Disease condition | |
| Hypertension | Clinician Assessment: measured systolic BP/diastolic BP>140/90 or on intervention. |
| Type 2 diabetes | Clinician Assessment: measured fasting glucose ≥ 7 mmol/L (126 mg/dL) or on treatment. |
| Tuberculosis | Self-report: Ever been told that you have TB in the past 12 months? |
| HIV infection | Self-report: Ever been told that you have HIV? |
| Cardiovascular disease | Self-report: Ever been told that you have cardiovascular disease? ie stroke, angina, heart attack or congestive heart failure |
| High cholesterol | Self-report: Ever been told you have high cholesterol? |
| Kidney disease | Self-report: Ever been told you have kidney disease? |
| Cancer | Self-report: Ever been told you have breast, cervical, prostate or other cancers? |
| Asthma/reactive air disease | Self-report: Have had asthma or reactive air disease? |
| | Self-report: Have had ulcers? |
| Depression | Self-report: Ever been told that you have TB in the past 12 months? |
| Arthritis | Self-report: Ever been told that you have arthritis in the past 12 months? |
| Chronic neuromuscular disease | Self-report: Ever been told that you have chronic neuromuscular disease? |
| Obesity | Measured: Body mass index (weight (kg)/height ($m^2$))≥30kg/$m^2$ |
